# Age-dependent mating dynamics, body size, and sex ratio in colonised *Anopheles coluzzii*

**DOI:** 10.1016/j.crpvbd.2026.100431

**Published:** 2026-09-03

**Authors:** Manuela Carnaghi, Kithmali Gayathri Senevirathna, Stephen Young, Frances Madeline Hawkes

**Affiliations:** a Natural Resources Institute, University of Greenwich, Chatham, ME4 4TB, UK; b Department of Medical Microbiology, Radboud University Medical Centre, Nijmegen, the Netherlands

**Keywords:** Mosquitoes, Insemination, Wing length, Body weight, Malaria vector, Laboratory colony

## Abstract

Malaria is a serious life-threatening disease transmitted by *Anopheles* mosquitoes. Knowledge of physiological parameters associated with mosquito fitness is essential to understand mating dynamics, population structure, and the effectiveness of vector control interventions. This study investigated key biological parameters, including body size and insemination rate in relation to adult age, as well as sex ratio, in a laboratory-reared *Anopheles coluzzii* colony. A total of 1526 female mosquitoes were dissected, of which 1494 were included in the analysis; a significant non-linear relationship was found between age and insemination status (Quasibinomial GLM, *F*_(1,48)_ = 265.3, *P* < 0.001). Insemination rates of 1-day-old females were low but not absent (mean ± SEM: 6.00% ± 2.5%); at three days, insemination rates were highly variable, ranging from 13.33% to 86.67%, suggesting inconsistent mating success, while the highest insemination rates were observed in older females. Morphometric analyses indicated that females presented significantly longer wings (mean ± SEM: 3.16 ± 0.01 mm) than males (mean ± SEM: 2.91 ± 0.01 mm), and that mosquitoes sampled at older ages had larger average wing lengths (*F*_(1,57)_ = 5.60, *P* = 0.02). Moreover, a significant male-biased sex ratio was found, with males representing on average 52.0% (95% CI: 50.54–53.66%) of the emerged adults. Together, these findings provide detailed baseline data on age-dependent mating success, body size variation, and sex ratio structure in laboratory-reared *An. coluzzii*.

## Highlights

• Insemination increased sharply during the first 5 days post-emergence.
• Early mating suggests rapid male sexual maturation in some individuals.
• Morphometric traits differed significantly between sexes.
• Larger mosquitoes were more common at later adult ages.
• The colony showed a male-biased sex ratio.

## Graphical abstract

**Figure:**
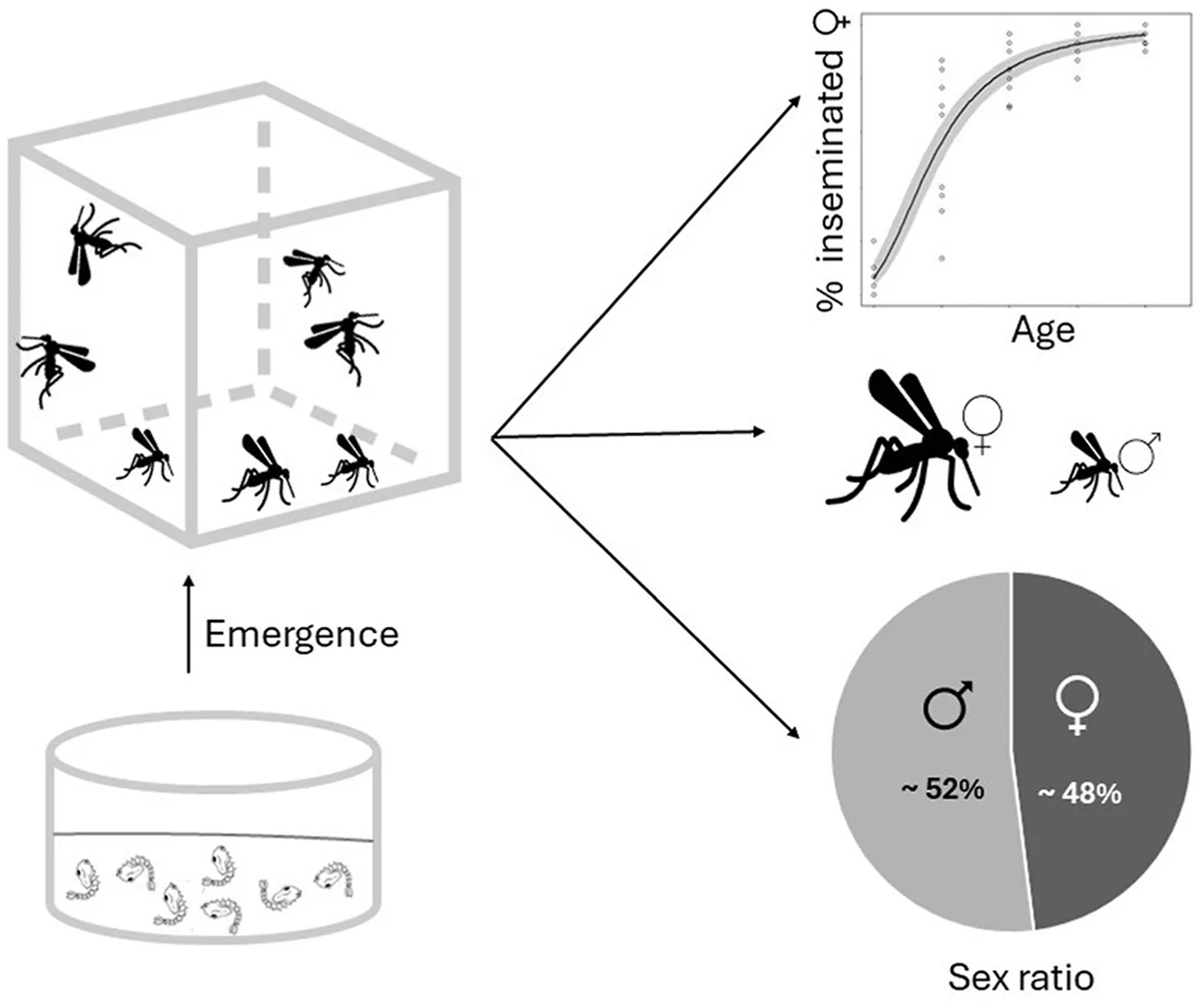

## 1 Introduction

Malaria remains the most widespread vector-borne disease, causing over 400,000 deaths annually, primarily among children under five and pregnant women (Adams, 2021; WHO, 2024a). In 2021, malaria was endemic in 85 countries, with sub-Saharan Africa accounting for 95% of global cases (WHO, 2021). Despite reductions since the early 2000s, in 2023 incidence increased to 263 million cases, with rates rising from 58.6 to 60.4 per 1000 population at risk (WHO, 2024b). Malaria is transmitted by ∼40 species of anopheline mosquitoes, which act as vectors for the protozoan parasites *Plasmodium* spp. when taking blood meals (Foster et al., 2017; Asmare et al., 2022). *Anopheles coluzzii*, a highly endophagic, endophilic, and anthropophilic species, is the primary vector in Central and West Africa, including Burkina Faso, Mozambique, Ghana, Mali, Benin, Nigeria, Gabon, Liberia, and Cameroon (Wiebe et al., 2017). This mosquito species plays a major role in malaria transmission during the dry season, as its larvae can survive and develop in temporary breeding sites (Akogbéto et al., 2018). These behavioural and ecological traits contribute to its persistence and reinforce its significance as a malaria vector.

Reproductive fitness is a key determinant of mosquito population dynamics and has direct implications for disease transmission and vector control (Helinski and Harrington, 2011). Following insemination, female mosquitoes undergo physiological and behavioural changes (Ramírez-Sánchez et al., 2023) that can affect when they initiate host-seeking, thus influencing their capacity to transmit pathogens (Alfonso-Parra et al., 2016). Understanding these processes is particularly important for novel vector control strategies, including the Sterile Insect Technique and gene drive techniques, where mating success is a primary factor determining the efficacy of population suppression (Alphey, 2014). Successful implementation of these methods requires that released males effectively compete with wild males to inseminate females, thereby reducing the reproductive output of the population and ultimately limiting disease transmission. Among the traits influencing reproductive fitness, body size was found to be an important factor in several mosquito species and can be taken as a proxy indicator of overall fitness (Menge et al., 2005). For example, in *Anopheles arabiensis*, larger females were shown to produce higher numbers of eggs and were recorded to have longer survival (Ameneshewa and Service, 1996). Similarly, in *Culex pipiens*, larger females were recorded to have higher fecundity (Villarreal et al., 2025), larger *Anopheles gambiae* males had greater copulatory success than smaller males (Sawadogo et al., 2013), and in *Wyeomyia smithii* mosquitoes, smaller males were found to be less successful in siring offspring compared to the larger males (Benjamin and Bradshaw, 1994). Wing length is the most widely used proxy for estimating mosquito body size (Yaro et al., 2012), given the strong positive correlation between the two traits (Niang et al., 2020). Mosquito body size is influenced by environmental temperature (Zubair et al., 2021), sex (Zubair et al., 2021), season (Faiman et al., 2020), and the nutrition and diet of the larval stages (Kulma et al., 2013). Adult body size varies significantly between laboratory-reared and field-collected individuals of *An. gambiae* (*sensu lato*), with wild males being ∼17% larger than laboratory-reared individuals (Huho et al., 2007). This considerable variability in body size, even within the same species, reinforces the importance of basic studies that examine physiological and morphological parameters in different strains or species. Furthermore, because laboratory-reared colonies are extensively used in experimental studies and in the development of vector control strategies, including those relying on mass rearing and release, it is essential to characterize how key fitness-related traits such as body size are expressed under laboratory conditions, in order to accurately interpret experimental results and evaluate their relevance to wild populations.

Another major determinant of reproductive fitness in mosquitoes is adult age (Sawadogo et al., 2013). After emergence, anopheline mosquitoes typically require a period of 24 h to reach sexual maturity, during which mating is generally unsuccessful, largely because males must undergo a 180° rotation of the genitalia before successful copulation is possible (Oliva et al., 2011). The timing of peak male mating activity varies among *Anopheles* species, with, for example, *Anopheles stephensi* reported to peak at three days of age (Mahmood and Reisen, 1982), whereas *An. gambiae* (*s.l.*) and *Anopheles culicifacies* peak at seven days post-emergence (Mahmood and Reisen, 1994; Verhoek and Takken, 1994). However, a study on *An. arabiensis* demonstrated that laboratory strains might exhibit accelerated sexual maturation, as some males in a laboratory colony were able to mate successfully as early as 11 h post-emergence (Oliva et al., 2011), highlighting the need to establish species- and strain-specific benchmarks for sexual maturity, including for laboratory-reared *An. coluzzii*. In females, reproductive performance is likewise age-dependent, with insemination success and fecundity reaching a peak before progressively declining with increasing age (Gabrieli et al., 2014; Ramírez-Sánchez et al., 2023). In contrast, field data from *An. gambiae* (*sensu stricto*) suggest that older male mosquitoes retain the ability to swarm and disperse, as their internal nutrient reserves increased with age (Huho et al., 2007), thus suggesting that male reproductive performance may not follow the same declining pattern described for females. Despite the epidemiological importance of *An. coluzzii* and its extensive use in laboratory-based studies, age-dependent patterns of insemination and associated fitness traits remain largely uncharacterized for this species. Therefore, the present study aims to characterize key physiological and reproductive parameters in a young laboratory colony of *An. coluzzii*, including the sex ratio, the probability of female insemination across post-emergence ages, and the relationship between body size, age, and sex. As these parameters directly influence mating behaviour and reproductive success, and consequently, population dynamics, a better understanding of their variation is essential and may provide valuable insights for the interpretation of laboratory studies as well as important information to be used in vector control strategies.

## 2 Materials and methods

### 2.1 Mosquito colony

Experiments were conducted on an*. coluzzii* colony which originated from a colony established from wild gravid females collected in Vallée du Kou, Burkina Faso, in 2017. At the time of the experiments, the colony had been maintained under laboratory conditions for ∼60 generations. Mosquitoes were reared following established protocols (Carnaghi et al., 2024). Particular care was taken to provide a consistent and adequate quantity of food during the larval stages, as food availability during this period can influence key fitness and physiological parameters (Kulma et al., 2013).

### 2.2 Experimental rearing conditions

Pupae were sexed based on morphological differences in the terminal abdominal segment (Papathanos et al., 2018) following established protocols (Leite et al., 2024), and exactly 100 male and 100 female pupae were placed together in a single cage and maintained together until dissection. This ensured a 1:1 adult sex ratio at the start of experiments and constant access to the opposite sex for mating. The pupal dish was left in the cage for 36 h, an interval identified as optimal to guarantee eclosion from all pupae based on preliminary studies conducted on our colony and prior studies on *An. gambiae* (Reiter and Jones, 1976; Leite et al., 2024), while also ensuring synchronized adult age within the cage. Observations conducted during preliminary work indicated that the majority of adults emerged within a ∼6-h window, occurring between 24 and 30 h post-pupal collection. Pupal collection was always performed at the same time of day, further standardising the age of adults within and across cages. Pupae were collected, sexed, and separated ∼1 h before scotophase, thus allowing two full dark cycles for emergence, as eclosion at ∼24–27 °C peaks during the dark phase (Reiter and Jones, 1976; Leite et al., 2024).

### 2.3 Mating success

Insemination status of adult females was determined at five time-points: 1, 3, 5, 7, and 9 days post-emergence. At each time-point, females were randomly selected, anesthetised with carbon dioxide, and placed on a microscope slide with a drop of physiological saline solution, prepared as described elsewhere (Hayes, 1953). Mosquitoes were dissected using entomological pins following established protocols (Benedict, 2015), and the spermathecae were examined for the presence of sperm bundles. Females with visible sperm in the spermatheca were considered inseminated; those without were classified as non-inseminated (Zubair et al., 2021). For each time-point, ten independent cohorts, i.e. ten cages, were analysed, and for each cohort, dissections were carried out until the insemination status of 30 females was determined.

### 2.4 Body size

Wing length was used as a proxy for mosquito body size (Yaro et al., 2012). Mosquitoes were sampled from the same groups used in the mating success experiment and at the same time-points: Day 1, 3, 5, 7, and 9 post-emergence. Individuals were randomly selected, placed in individual vials, and euthanised by freezing at −20 °C, where they were also stored until measurement. Only live mosquitoes at the time of sampling were selected to avoid dehydration-related size distortion. For measurement, each specimen was placed on a microscope slide, and the right wing was detached at the base using an entomological pin. If the right wing was absent or damaged, the left wing was used instead. Wings were examined under a stereomicroscope (Ultra ZOOM-2 ULWD, GT Vision, Suffolk, UK) paired to the GT Vision GXCapture-T software version x64, 3.7.13814.20190120 (GT Vision, Suffolk, UK). Wing length was measured as the distance between the alular notch and the distal tip to the nearest 0.01 mm (Yaro et al., 2012; Baeshen et al., 2014). Each measurement was taken in triplicate and the average recorded. Female wing length was measured at Days 3 and 7 post-emergence, as individuals were also concurrently sampled for insemination experiments, while males, which were not used in insemination dissections, were available in sufficient numbers for measurement across all five time-points and were therefore sampled across all time-points. Ten cohorts, i.e. cages, were assessed on Days 1, 5, and 9 post-emergence; eight groups were assessed on Days 3 and 7 post-emergence. For both sexes, 15–17 individuals per group were measured, with the only exception of one group of 3-day-old females, for which only eight mosquitoes were sampled. Body weight was assessed in males only, as females were concurrently used for insemination dissections; males aged 3 and 7 days post-emergence, with 8 and 7 groups sampled, respectively. For each group, 30 live male mosquitoes were randomly selected, dried for 48 h at 40 °C, weighed individually in triplicate on an analytical balance (AT201, Mettler Toledo), and the average was recorded. In one group on Day 3, only 10 mosquitoes were weighed.

### 2.5 Sex ratio

In parallel to the mating and body size experiments, the sex ratio of emerging adults was recorded. To do this, groups of individuals from the same cohort were reared under identical conditions but in separate cages: one set for the mating and body size experiments, and another for the sex ratio assay. This ensured that all mosquitoes belonged to the same generation, while avoiding the imposed 1:1 sex ratio used in the other assays, where pupae were manually selected. To do so, at the egg collection stage, half of the eggs were randomly selected for the mating and body size experiments, while the other half were used for sex ratio determination. Eggs were sampled from both spatially distant and clustered areas on the filter paper to ensure a representative mix. For each cohort, ∼250 ± 10 eggs were counted and placed in a rearing tray, where they were kept through the aquatic stages until pupation. Trays were checked every 12 h, and all pupae were transferred into a cylindrical emergence pod (Bioquip, Rancho Dominguez, USA) without the lid and placed in a cage. This allowed emerged adults to exit the water safely while minimising mortality. Pods were kept in the cage for 5 days after the final addition of pupae, or until all had emerged, whichever was the latter. Each cage, treated as a replicate, thus contained ∼200 randomly selected individuals from the same cohort. One cage for sex ratio assessment was set up for each cage used in the mating success experiment, i.e. for a total of 50 cages. Five days after the final pupae were introduced, or once all pupae had emerged, mosquitoes were euthanised by placing the cage at −20 °C for 1 h, then sexed and counted. This procedure allowed the inclusion of any individual that had emerged but died prior to the collection point.

### 2.6 Statistical analysis

Data analysis was conducted in R (v.4.4.3) (R Core Team, 2021) using the *MASS* package (Venables and Ripley, 2002) and base R functions for analysis of variance, analysis of covariance and regression models for wing length and weight data, and for generalised linear models (GLM) for proportion inseminated. The proportion of inseminated females and days post-emergence was assessed using a GLM, with a logit link and quasibinomial error distribution, as the binomial GLM was severely overdispersed. The logistic regression line was linear when the time predictor was log_e_-transformed; 95% confidence intervals around the regression line were calculated from the standard errors predicted by the GLM. The analysis of deviance used F-tests, with the dispersion parameter serving as residual mean deviance. Deviations from a sex ratio of 1:1 were tested using a null GLM with a logit link and quasibinomial errors. Under the logit transformation, a 1:1 ratio corresponds to 0; thus, a *t*-test on the model coefficient (the logit-scale average sex ratio) was used to determine whether the observed value significantly deviated from the 1:1 ratio. Logit-scale 95% confidence intervals were calculated from the predicted standard error of the mean, and the bounds were back-transformed to give natural sex ratios. Differences in wing length across mosquito sexes and post-emergence ages were analysed using a linear model and an analysis of covariance, with time as an x-variate and sex as a categorical independent factor. Effect sizes for the wing length model were calculated as partial eta-squared (*η*^2^). No sex-by-age interaction term was fitted in the wing length model, given the unbalanced sampling across sexes and ages. A linear regression assessed the relationship between male weight and wing length, and an analysis of variance (ANOVA) examined the difference in average male weight across post-emergence age. In all morphometric analyses, individual mosquitoes within each cage were measured to calculate the cage-level mean, and these means were treated as the unit of replication. Thus, the sample sizes reported reflect the total number of individuals measured, while the degrees of freedom reflect the number of independent groups. For all statistical analyses, the criterion for statistical significance was defined as *P* < 0.05. Model validation used the Shapiro-Francia test, R package *nortest* (Gross and Liggers, 2015), to assess whether the distribution of the models’ standardised residuals did not significantly differ from normality, both for linear models and GLMs. As the Shapiro-Francia test gave non-significant results (Table 1), the assumption of normality was considered satisfied, supporting the validity of the chosen models.

**Table 1:** Shapiro-Francia normality tests for model validation.

Description of tested variables	Statistical model used for testing variables	Results of the Shapiro-Francia normality test, using standardised residuals from the statistical model
Proportion of inseminated females *versus* post-emergence age	Quasibinomial GLM with logit link	*W* = 0.962, *P* = 0.095
Sex ratio	Null quasibinomial GLM with logit link	*W* = 0.975, *P* = 0.328
Male weight *versus* male wing length	Linear regression model	*W* = 0.958, *P* = 0.637
Male weight *versus* post-emergence age (3- and 7-day-old)	Linear ANOVA model	*W* = 0.9351, *P* = 0.275

## 3 Results

### 3.1 Mating success

**Fig. 1:**
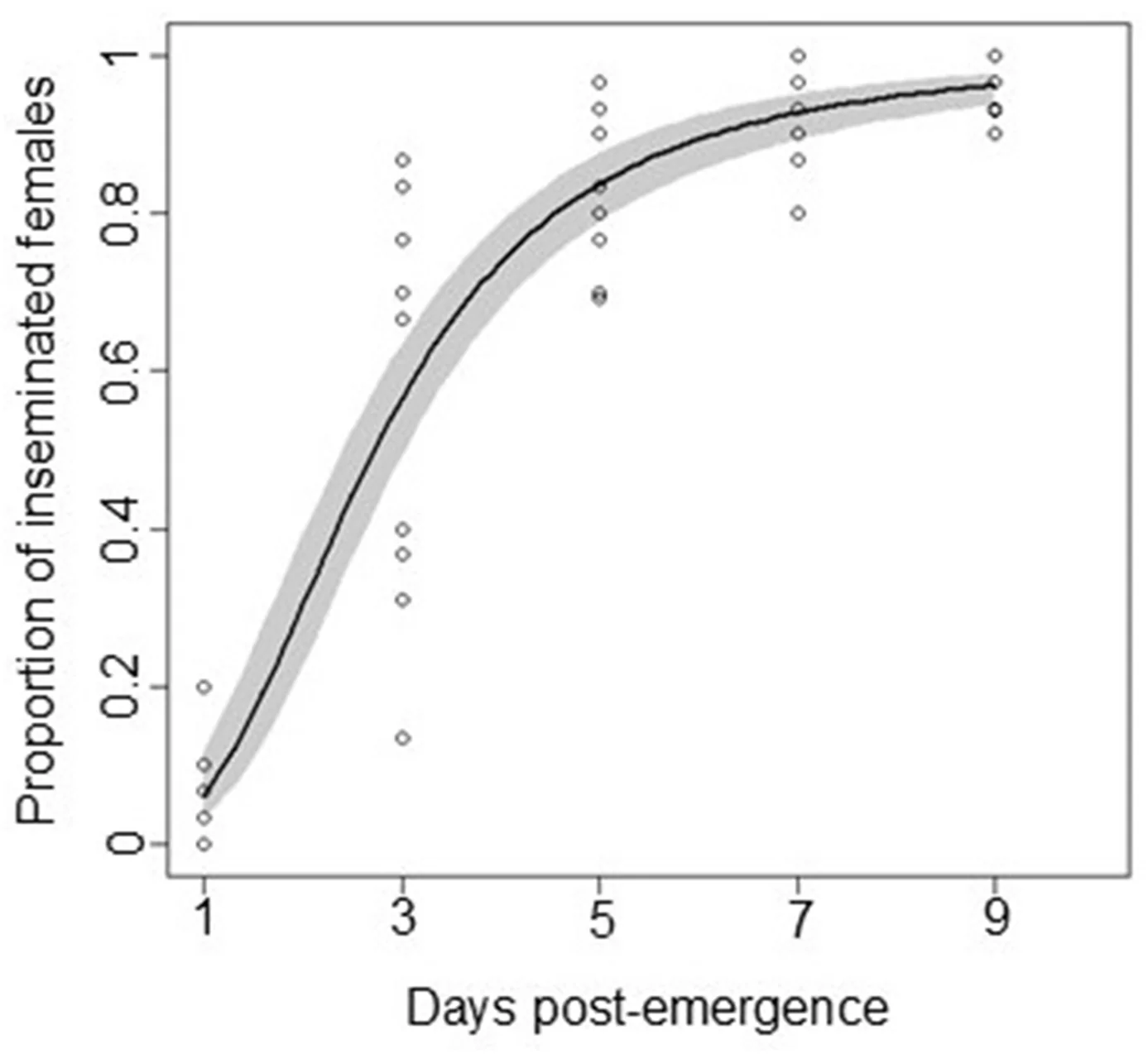
Insemination proportion over days post-emergence. The circles represent results for individual cages; predicted logistic regression curve indicates the relationship between the age and the proportion of inseminated mosquitoes; the grey band indicates the 95% CI. The insemination trend on the post-emergence age is highly significant (analysis of deviance, quasibinomial GLM with logit link, *F*_(1,48)_ = 265.3, *P* < 0.001).

A total of 1526 female mosquitoes were dissected to determine their insemination status. Of these, insemination status in 32 individuals could not be differentiated, and these were excluded from the analysis; thus, the analysis of the insemination status was conducted on the results obtained from 1494 females. Ten cages were assessed for each time-point, and insemination status was determined for 30 females per cage, except for one cage on Day 3, one on Day 5, and one on Day 9, where 29, 26, and 29 females were successfully dissected, respectively. A significant relationship was found between the post-emergence age and the proportion of insemination (*F*_(1,48)_ = 265.28, *P* < 0.001), with the likelihood of finding an inseminated female increasing as the age of the population increased (Fig. 1). Notably, a steeper increment of insemination proportion was found in the early time-points post-emergence, with the increment slope decreasing after Day 5, by which ∼80% of the females in a cage are expected to be inseminated. Females aged one day post-emergence exhibited the lowest insemination rate (mean ± SEM: 6.00% ± 2.5%), followed by those aged three days (mean ± SEM: 57.43% ± 5.2%), and five days (mean ± SEM: 83.26% ± 3.94%). Insemination rates on Day 3 showed considerable variability between cohorts, ranging from 13.33% to 86.67%, suggesting inconsistent mating success at this age, with some cages achieving insemination levels comparable to those observed on Day 5, while others showed markedly lower rates. The highest insemination rates were observed in females aged seven (mean ± SEM: 91.00% ± 3.02%) and nine (mean ± SEM: 97.31% ± 1.71%) days post-emergence.

### 3.2 Body size

**Fig. 2:**
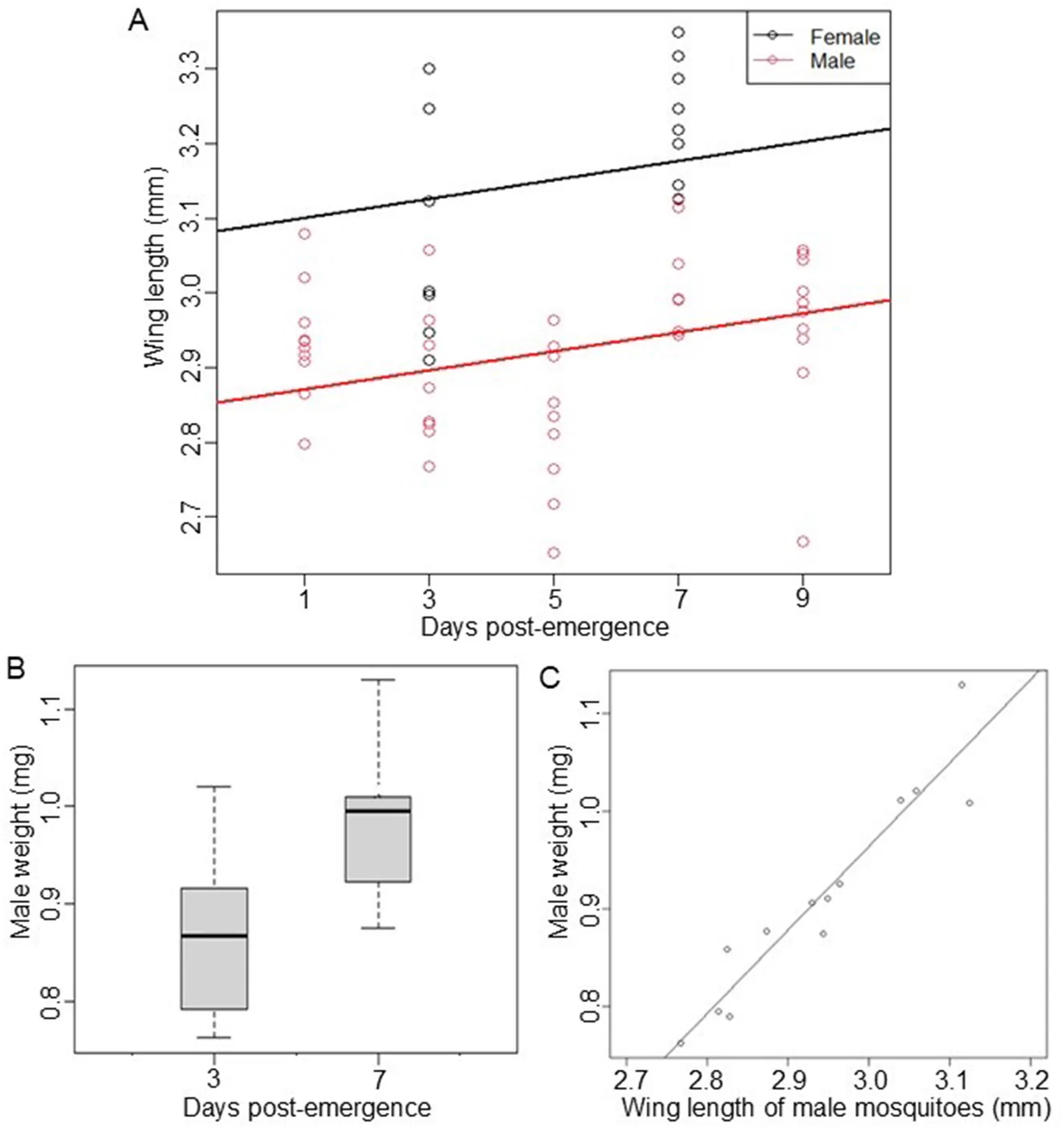
Morphometric analysis of *An. coluzzii* body parameters at different adult ages. **A** Wing length (mm) of males and females measured at different days post-emergence. Individual mosquito measurements were aggregated within each cage, and cage-level means were used as the experimental units for statistical analysis. **B** Dry body weight (mg) of males at three and seven days post-emergence; bold black lines indicate the median, upper and lower limits of the box indicate the interquartile range, whiskers indicate the maximum and minimum data points. **C** Scatter plot showing the relationship between the body weight (mg) and wing length (mm) of males, with a fitted linear regression line indicating the correlation (*R*^2^ = 0.89, fitted regression equation: weight = wing length*0.85704 – 1.60734).

The wing length was measured in 168 males aged 1-day-old, 130 males aged 3-day-old, 170 males aged 5-day-old, 113 males aged 7-day-old, and 161 males aged 9-day-old, for a total of 742 male individuals recorded. For females, a total of 265 individuals were measured, with 129 being 3-day-old and 136 being 7-day-old. Results revealed a strong and significant effect of sex on the wing length (*F*_(1,57)_ = 45.63, *P* < 0.001, partial *η*^2^ = 0.445), with females exhibiting significantly larger wing lengths (mean ± SEM: 3.16 ± 0.01 mm) than males (mean ± SEM: 2.91 ± 0.01), corresponding to an absolute difference of ∼8.6%. Results further indicate that post-emergence age also had a significant effect on the wing length, though smaller in magnitude (*F*_(1,57)_ = 5.60, *P* = 0.02, partial *η*^2^ = 0.089), with individuals sampled at older ages exhibiting larger wings compared to those at younger ages (Fig. 2A). Dry body weight was recorded for 430 male mosquitoes, including 220 individuals at 3 days post-emergence and 210 at 7 days post-emergence. A one-way ANOVA revealed a significant difference in the dry body weight across the two different ages (*F*_(1,13)_ = 6.7, *P* = 0.022) (Fig. 2B). Male mosquitoes sampled 7 days post-emergence were found to be heavier (mean ± SEM: 0.98 ± 0.03 mg) compared to those sampled at 3 days post-emergence (mean ± SEM: 0.87 ± 0.03 mg). A linear regression analysis revealed a significant positive correlation between wing length and dry body weight of male mosquitoes (*F*_(1,11)_ = 96.86, *P* < 0.001, *R*^2^ = 0.89) (Fig. 2C). This relationship suggests a proportional growth pattern between body mass and wing length, with larger wing lengths associated with greater body masses. In summary, these results indicated that *An. coluzzii* adult females are larger than their male peers, and mosquitoes sampled at older ages tend to be larger than those sampled at younger adult ages.

### 3.3 Sex ratio

The number of male and female individuals that reached the adult stage was successfully measured for 48 out of the 50 cages. Data from two cages were excluded due to unfavourable laboratory conditions that precluded reliable assessment. Of the ∼250 eggs used for each cohort, on average, 215.18 ± 36.11 (SD) were recovered as adults. In total, 10,012 mosquitoes were successfully sexed, 5216 being male and 4796 being female, while for 317 (3% of the total number of mosquitoes recovered in the 48 cages) it was not possible to differentiate the sex due to the loss of key morphological features. The recorded sex ratio significantly deviated from the expected ratio of equal numbers for both sexes (*t* = 2.71, *df* = 47, *P* = 0.009, test that the null model quasibinomial GLM coefficient is non-zero). There were significantly more male adults, on average 52.00% (95% CI: 50.54–53.66%), than the expected 50%. The estimated mean percentage of females was 47.90% (95% CI: 46.34–49.46%).

## 4 Discussion

Understanding the factors that determine the timing and physiological responses associated with mating in mosquito colonies is critical for ecological studies and vector control interventions, as mating can directly influence female fecundity and longevity (Howell and Knols, 2009; Oliva et al., 2013), and thus indirectly, population dynamics. The present study demonstrates a significant positive, non-linear relationship between post-emergence age and the proportion of inseminated *An. coluzzii* females, with insemination increasing steeply in the first five days post-emergence, exceeding 91% by Day 7 before approaching an asymptote at around Day 9, where it reached ∼97%.

Age plays a central role in influencing reproductive outputs in insects (Grotewiel et al., 2005; Miller et al., 2014), with older females tending to produce fewer eggs of reduced viability; a pattern seen across a wide range of insect taxa (David et al., 1975; Fricke et al., 2013; Tabata and Teshiba, 2018; Tasnin et al., 2021). Male age has also been shown to influence insemination outcomes. In the present study, insemination rates in *An. coluzzii* remained relatively low and highly variable at Day 3 (∼57.43%) before increasing sharply at later time-points. A similar age-dependent increase in mating success has been reported across members of the *An*. *gambiae* complex. In *An. coluzzii*, males achieved the highest mating success between four and eight days post-emergence (Sawadogo et al., 2013), while studies on *An. gambiae* (*s.l.*), *An. gambiae* (*s.s.*), and *An. arabiensis* consistently found that insemination rates increased progressively after emergence and generally peaked between five and seven days of age (Verhoek and Takken, 1994; Okal et al., 2015). Insemination rates, however, varied between species, peaking at ∼72% at Day 6 in *An. gambiae* (*s.s.*) and only at around 45% at Day 5 in *An. arabiensis* (Okal et al., 2015). Collectively, these findings suggest that sexual maturation occurs progressively over the first week post-emergence. Interestingly, we report higher insemination rates at earlier ages (∼83% at Day 5 and 91% at Day 7) compared to previous studies. Whether this difference reflects genuine interspecific variation, differences in rearing conditions, or strain-specific acceleration of sexual maturity (Oliva et al., 2011) warrants further investigation.

The low but non-zero insemination rate observed here at one day post-emergence merits particular attention, as it departs from the pattern reported for sister species within the *An. gambiae* complex. A study found no insemination in *An. arabiensis* colonies until the second night post-emergence, and none in *An. gambiae* (*s.l.*) until the fourth night (Verhoek and Takken, 1994). This difference can be contextualised through variation in timing of male genitalia rotation, which must be completed before successful copulation is possible (Oliva et al., 2011). The process is highly species-specific, ranging from 6 h in *Culiseta inornata* (Rees and Onishi, 1951) to up to 30 h in *Aedes taeniorhynchus* (Provost et al., 1961). Within anophelines, rotation was reported to take ∼20 h in *An. stephensi* and *An. arabiensis* (Mahmood and Reisen, 1982; Oliva et al., 2011), and up to 36 h in *Anopheles funestus* (Dahan and Koekemoer, 2014). Given that in the present study mosquitoes had access to only one full dark cycle before the one-day dissection time-point, the detection of inseminated females at this age suggests that a subset of males may have achieved functional sexual maturity within just a few hours of emergence, although this was not directly measured. This is consistent with the phenomenon of accelerated sexual maturation reported in a laboratory-reared colony of *An. arabiensis*, in which successful insemination was detected as early as 11 h post-emergence and attributed this to early completion of genitalia rotation in their laboratory strain (Oliva et al., 2011). Accelerated maturation represents one plausible explanation for the early insemination observed here. *Anopheles* mosquitoes typically mate in aerial swarms; however, swarm formation and consequent mating success are known to be altered in confined laboratory environments (Hammond et al., 2021). Thus, the gradual increase in insemination observed here may reflect the progressive establishment of successful mating interactions within cages, rather than delayed physiological readiness to mate. The considerable inter-cage variability at Day 3 (13.33–86.67%) further suggests that the transition through sexual maturity is not synchronous across individuals or cohorts. Characterising insemination dynamics is essential as maturation timing is a species-specific trait (Oliva et al., 2011; Djègbè et al., 2024), and even modest differences could alter the outcomes of interventions that depend on competitive mating. As this colony has been maintained under controlled conditions since 2017, laboratory adaptation may have influenced the rate of sexual maturation, and comparative studies in recently field-collected *An. coluzzii* would be valuable to determine how representative these findings are of wild populations.

Sexual dimorphism in mosquitoes is a well-established phenomenon and includes a larger female body size, driven by growth-regulating genetic traits (Benjamin and Bradshaw, 1994; Duman-Scheel and Syed, 2015; Barr et al., 2023). This size difference has important functional consequences, as females possess greater lipid reserves, which are essential for egg development and sustained flight (Meuti and Short, 2019; Pinch et al., 2021) required during host-seeking over long distances (Traoré et al., 2021). Larger body size also correlates with enhanced reproductive capacity, including higher follicle counts and larger blood meal storage volume (Lyimo and Takken, 1993; Yaro et al., 2006), and it is positively selected during mating (Okanda et al., 2002; Zubair et al., 2021). Consistent with these established patterns, females from our colony presented significantly longer wings than males, in line with previous observations across multiple culicid species (Koella and Lyimo, 1996; Fernandes and Briegel, 2005; Loetti et al., 2011; Fouet et al., 2012; Yeap et al., 2013; Barreaux et al., 2018; Garzón and Schweigmann, 2018; Traoré et al., 2021; Maiga et al., 2025; Ottih et al., 2025). It should be noted that in the present study body weight was assessed in males only; thus, sexual dimorphism in body mass was not quantified, and body size comparisons were conducted mostly on wing length measurements. Interestingly, mosquitoes sampled at older adult ages had a slightly larger body size compared to those sampled at younger ages, and this was consistent across sexes. Several studies have reported unchanged wing size for *An. gambiae* mosquitoes across different ages, recorded from one to five days post-emergence (Okanda et al., 2002; Hariharan et al., 2016; Barr et al., 2023), and it was found that in males and females of *An. gambiae*, the wing dimensions stabilise within 24 h post-emergence (Takken et al., 2013). In contrast, a transient 1.5–2% increase in wing length during the first five days post-emergence was reported in *An. stephensi* (Takken et al., 2013) and was attributed to wing membrane stretching during delayed cuticular sclerotization (Takken et al., 2013; Hanna et al., 2023; Salcedo et al., 2023). The increases observed here (∼1.02% in males from Day 1 to Day 9; ∼5.19% in females from Day 3 to Day 7) are larger in magnitude and span a considerably longer period. Thus, we hypothesise that this pattern reflects the selective survival of larger individuals. Mosquito body size is positively associated with lipid stores in wild and laboratory-reared male *An. gambiae* (*s.l.*) (Huho et al., 2007), which may facilitate the greater survival rate observed in several species, including *An. arabiensis* (Ameneshewa and Service, 1996), *An. gambiae* (Lehmann et al., 2006), *Aedes sierrensis* (Hawley, 1985), *Aedes aegypti* (Mogi et al., 1996), and *Culex tarsalis* (Reisen et al., 1984). Thus, it is possible that the population sampled at later time-points was composed primarily of larger mosquitoes solely because those were more likely to have survived longer. Further studies that follow the lifespan and body size of individual mosquitoes are suggested to further elucidate the relationship between longevity and body size.

In this study, a strong linear correlation was found between body weight and wing size of male *An. coluzzii* mosquitoes, supporting the use of wing length as a proxy for body size (Koella and Lyimo, 1996). However, the relationship between wing length and body weight is not universal across mosquitoes, and has been shown to be species-, sex-, and rearing condition-specific (Nasci, 1990; Siegel et al., 1992). Additionally, this relationship can differ among specimens reared in the laboratory *versus* those collected from the field (Nasci, 1990), thus reinforcing the necessity of characterising these relationships across different species and under different rearing conditions.

Beyond individual fitness traits, population-level characteristics such as sex ratio can substantially affect mosquito control outcomes. In *Anopheles* mosquitoes, a balanced 50:50 sex ratio is generally considered the expected norm (Oliva et al., 2011) and has been consistently reported across multiple species and experimental contexts (Horosko et al., 1997; Grieco et al., 2003; Epopa et al., 2018; Akpodiete and Tripet, 2021; Zengenene et al., 2021; Zubair et al., 2021; Puchot et al., 2022). However, some reports of a deviation from a 50:50 sex ratio skewed towards males have been recorded in colonies of *An. gambiae* (Simoni et al., 2020), *An. stephensi* (Yadav et al., 2017), *Cx. pipiens* (Sweeny and Barr, 1978), and *Ae. aegypti* (Hickey and Craig Jr, 1966), consistent with the male-biased ratio observed in the present study. From an applied perspective, the induction of strongly male-biased sex ratios has been proposed as an effective method to suppress or eliminate pest populations (Galizi et al., 2014). A male-biased sex ratio could in principle benefit mass-rearing programmes by increasing male yield. The ∼2% deviation observed here, while statistically significant, is biologically modest and it is unlikely to confer substantial practical advantages. Nevertheless, the mechanisms underlying these deviations remain of interest, as elucidating them may yield insights relevant to programmes actively seeking to optimise male-biased production.

## 5 Conclusions

The present study describes key biological parameters of colonised *An. coluzzii*, including body size dimorphism, insemination rates, and adult sex ratio, which are crucial for understanding mosquito fitness and population dynamics. The findings show that the proportion of inseminated females increases with the age of the population; surprisingly, some mating even occurred within one day of emergence. The sex composition of the studied colony deviated from a 50:50 ratio, with more males being recorded in the population; female mosquitoes were larger than male mosquitoes, and mosquitoes sampled at older ages had greater wing length and weight compared to those sampled at younger post-emergence ages. Importantly, the age-dependent insemination profile and the pronounced inter-cage variation at Day 3 provide a reference framework for future studies investigating sexual maturation and reproductive performance in *An. coluzzii*. These results emphasise the importance of considering species-specific traits, as modest variabilities between species could influence the outcomes of control interventions.

## CRediT authorship contribution statement

**Manuela Carnaghi:** Conceptualization, Methodology, Investigation, Data curation, Formal analyses, Visualization, Project administration, Supervision, Writing - original draft, Writing - review & editing. **Kithmali Gayathri Senevirathna:** Investigation, Data curation, Writing - original draft, Writing - review & editing. **Stephen Young:** Formal analyses, Visualization, Writing - review & editing. **Frances Hawkes:** Conceptualization, Supervision.

## Ethical approval

Not applicable. This study was conducted in accordance with the following guidelines for animal welfare and/or reporting: UK National Guidelines. Ethics committee approval was not required. The research involved only invertebrate vector organisms and did not involve human participants, vertebrate animals, or endangered species.

## Statement on the use of AI-assisted technologies

The authors declare that no artificial intelligence (AI) technologies were used in the creation of this manuscript.

## Funding

This research was supported by internal funding from the Behavioural Ecology Research Group, Natural Resources Institute, 10.13039/100010027University of Greenwich, Chatham, UK.

## Declaration of competing interest

The authors declare that they have no known competing financial interests or personal relationships that could have appeared to influence the work reported in this paper.

## Data Availability

The data supporting the conclusions of this article are included within the article. The data collected and analyzed during the study are available from the corresponding author upon request.
